# Measuring visual information gathering in individuals with ultra low vision using virtual reality

**DOI:** 10.1038/s41598-023-30249-z

**Published:** 2023-02-23

**Authors:** Arathy Kartha, Roksana Sadeghi, Chris Bradley, Chau Tran, Will Gee, Gislin Dagnelie

**Affiliations:** 1 Department of Ophthalmology, Johns Hopkins University School of Medicine, Baltimore, MD USA; 2 Department of Biomedical Engineering, Johns Hopkins University, Baltimore, MD USA; 3 BaltiVirtual Inc., Baltimore, MD USA

**Keywords:** Diseases, Health care, Medical research

## Abstract

People with ULV (visual acuity ≤ 20/1600 or 1.9 logMAR) lack form vision but have rudimentary levels of vision that can be used for a range of activities in daily life. However, current clinical tests are designed to assess form vision and do not provide information about the range of visually guided activities that can be performed in daily life using ULV. This is important to know given the growing number of clinical trials that recruit individuals with ULV (e.g., gene therapy, stem cell therapy) or restore vision to the ULV range in the blind (visual prosthesis). In this study, we develop a set of 19 activities (items) in virtual reality involving spatial localization/detection, motion detection, and direction of motion that can be used to assess visual performance in people with ULV. We estimated measures of item difficulty and person ability on a relative d prime (*d*′) axis using a signal detection theory based analysis for latent variables. The items represented a range of difficulty levels (− 1.09 to 0.39 in relative *d*′) in a heterogeneous group of individuals with ULV (− 0.74 to 2.2 in relative *d*′) showing the instrument’s utility as an outcome measure in clinical trials.

## Introduction

According to the most recent estimates reported by World Health Organization in 2020, there are 43.3 million people who are blind (VA < 20/400 or 3/60 or 1.3 logMAR) worldwide^1^. This includes people with ultra-low vision (ULV), who are in the lowest end of the low vision spectrum and whose estimated visual acuity ranges from bare light perception to 20/1600 (1.9 logMAR)^2^. A growing number of sight restoration treatments that are in development or in various stages of clinical trials recruit participants with ULV, including stem cell therapies, gene therapies, and visual prostheses that restore vision to ULV levels^3–14^. It is therefore important to develop outcome measures that quantify visual performance in people with ULV to assess the efficacy of vision restoration and rehabilitation programs.

How does one measure visual performance in people with ULV? Historically, visual acuity and/or visual field have been used to compare a person’s level of vision against reference standards. For example, 20/20 (0.0 logMAR) with a total horizontal field of 190° is considered normal vision, 20/40 (0.3 logMAR) with a binocular horizontal visual field ranging between 55 and 150° is the standard level required for driving across various states in the United States^15^, and ≤ 20/200 (1.0 logMAR) and/or visual field diameter ≤ 20° is the reference standard for legal blindness. In ULV, we cannot measure visual acuity or visual fields using standard techniques such as the ETDRS or Bailey-Lovie charts, or the Humphrey Field Analyzer for visual fields. A person with ULV cannot read the top line of letters on an ETDRS chart, even from the closest distance of 0.5 m which corresponds to 20/1600. A person with ULV may also not be able to see a Goldmann size ‘V’ target (1.72°) in a visual field test. The Berkeley Rudimentary Vision Test (BRVT)^16^, Freiburg Visual & Contrast Sensitivity Acuity Test (FrACT)^17^ and Basic Assessment of Light and Motion (BALM)^18^ were specifically designed to quantitatively estimate visual acuity down to light perception (LP) in people with ULV using a combination of letters (the single letter ‘E’ or Landolt C) and gratings. For instance, with the basic cards using black and white discrimination (BWD) and white field projection (WFP) in BRVT, it is possible to estimate up to 3.5 log MAR. However, using these tests, it is not possible to infer visual performance, i.e., how effectively a person with ULV uses vision in day-to-day life^19–21^.

What is the range of activities that can be performed with ULV? One way to assess this is through patient-reported outcome measures (PROMs) in the form of questionnaires. Most clinical trials currently use validated PROMs in order to satisfy regulatory requirements^22^. There are only two questionnaires specifically aimed at people with ULV: the impact of visual impairment in very low vision (IVI-VLV)^23^ and the ultra-low vision visual functioning questionnaire (ULV-VFQ)^24^. The IVI-VLV specifically addresses the ‘impact’ of very low vision (VA < 1.0 logMAR, visual field < 10°) while the ULV-VFQ quantifies the ‘visual ability’ of people with profound visual impairment or ULV (VA < 20/500 or 1.4 logMAR). The 150 items in the ULV-VFQ were selected from 760 activities reported by focus groups of individuals with ULV as visually relevant in their daily lives. Together these instruments can measure the perceived impact and ability among people with ULV. However, self-reports are necessarily subjective^25,26^, and there is the possibility that people will under or over-rate their disability or the impact of their visual impairment. Moreover, self-reports can also be dependent on non-visual factors such as depression, anxiety, and adjustment to vision loss^27,28^. Response bias also exists with observer-rated evaluation of visual performance in people with ULV such as the functional low vision observer-rated assessment (FLORA™), besides the potential Hawthorne effect^29^.

One visual performance test in low vision is MNREAD chart^30,31^, designed to evaluate reading performance across a range of high contrast print sizes at normal reading distance in people with low vision and widely used in clinical trials as a standard outcome measure^32–34^. However, people with ULV do not have form vision; therefore, any tasks involving detailed vision, such as reading, cannot be included in a visual performance assessment for ULV; visual information gathering, eye-hand coordination, and mobility have been identified as the functional domains that are most relevant to this population, according to a study by Adeyemo et al.^35^. The same study identified visual aspects such as contrast, luminance, ambient lighting, and size to be decisive factors for effective vision use in ULV. It is important to study the range of standardized activities in the three relevant functional domains that can be performed with ULV, so they can be used to assess the functional outcomes of various sight restoration and vision rehabilitation treatments to quantify the use of residual/restored vision^36^.

Ideally, visual performance on a range of tasks and luminance levels relevant in day-to-day life should be measured in the real world. However, measuring visual performance in the real world comes with its own challenges. For example, it is difficult to control ambient lighting and contrast when performing real world activities outside a clinical setting. As a compromise, one could assess real world visual performance in the clinic. However, it is cumbersome in a clinical setting to set up real world tasks that can be used for the assessment, and to standardize those tasks across settings, such as for clinical trials in multiple centers or in different parts of the world. In lieu of measuring performance of individuals with ULV in real world settings, virtual reality (VR) provides the next best alternative. Many different types of tasks based on activities of daily living can be presented in a controlled and standardized manner in VR.

The aims of this study are, for the visual information gathering domain, to: (1) develop a standardized VR-based assessment for individuals with ULV; (2) evaluate the psychometric properties of the assessment.

## Methods

### Overview

To select our performance measures we used items from ULV-VFQ that fall in the domain of visual information gathering and that lend themselves to a close equivalent in real-world activities. A set of 17 real-world activities were pilot tested in a small group of people with ULV (n = 24)^37^. In developing VR equivalents, we decided to add two scenes dealing with temporal processing since this is a little studied aspect of ULV. Our activities include 13 items based on spatial localization, 3 on the direction of motion, and 3 on flicker/movement detection because this distribution approximated the proportions in the ULV-VFQ. The scenes were presented inside a commercial virtual reality (VR) headset (FOVE Inc.) with a screen resolution of 1280 × 1440 pixels per eye and a frame rate of 70 Hz.

There were 3 difficulty levels for each item (i.e., task), which were created by modifying contrast, timing, or movement amplitude depending on item. The step sizes used for presenting different difficulty levels were large enough to cover the full range of ULV. For the spatial localization tasks, each scene was presented at three contrast levels (10%, 30%, and 100%). For the direction of motion tasks, the three levels were slow (2 s duration), medium (1 s), and fast (0.5 s) presentation times; for movement detection, there was an item with three flicker frequencies (3.5 Hz, 11.7 Hz, and 35 Hz) and another scene where movement amplitude of a soccer ball was changed. The total number of items, therefore, is 57 (19 × 3) when all difficulty levels are considered. The luminance levels inside the headset were measured using a Konica Minolta CS-100 spot photometer. The highest luminance (room light on scene) was measured to be 70 cd/m^2^ and lowest luminance 0.12 cd/m^2^. Contrast levels and grey scales used in the tasks were selected on the basis of performance in pilot experiments. For specific details about the stimuli, see Supplementary Document and Supplementary Table S1.

**Figure 1:**
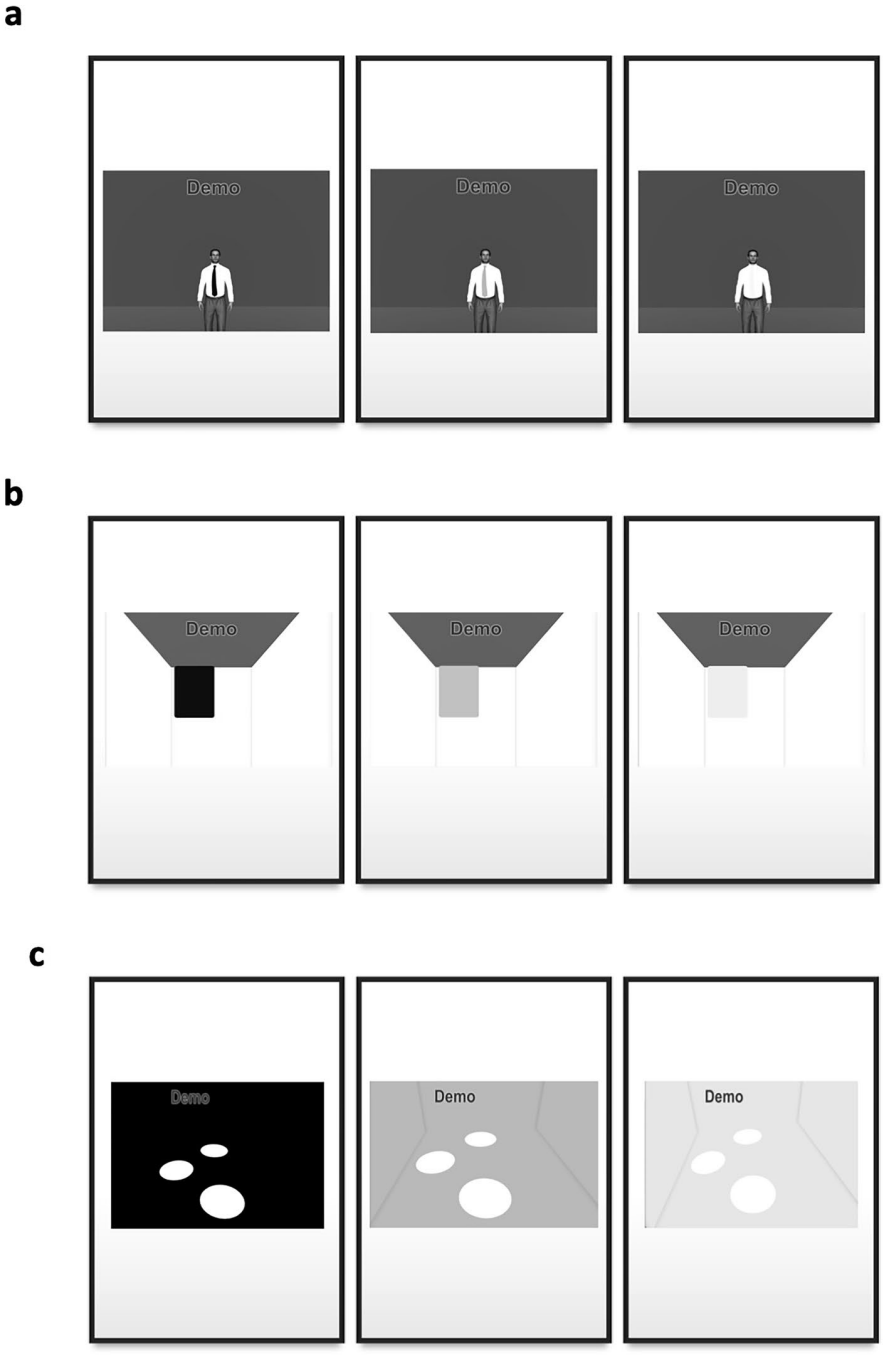
Examples of 2, 3 and 4-AFC spatial localization/detection tasks at 3 difficulty levels. (**a**) A 2-AFC task to detect whether a person is wearing a tie or not: black (high contrast), blue (medium contrast), and yellow (low contrast). (**b**) A 3-AFC task to detect whether a hanging towel is on the left, on the right, or missing. (**c**) A 4-AFC task to determine the location of the missing plate: 3, 6, 9, or 12 o’clock. For a complete set of tasks see the supplement.

All items were structured as $m$-alternative forced-choice tasks ($m$-AFC; $2\le m\le 4$), where on each trial there were $m$ possible response alternatives with one alternative defined as “correct”. Example detection/localization tasks for different $m$ can be seen in Fig. 1; the complete set of tasks is provided in the Supplementary Document. No auditory or tactile cues were provided for any of the tasks so that participants performed the tasks using only visual cues. Participants wore their habitual distance refractive correction and were allowed to move their heads freely to scan for objects in the virtual environment and use their preferred fixation. The angular subtense for all scenes was variable depending on the distance at which each participant viewed the scene which was at each individual’s optimal distance as they would do in the real-world. The size of the virtual room was 10 × 10 m and the size of the table was 0.8 × 0.8 m.

### Sample localization/detection tasks

Participants were asked to locate an object in each scene or report it as missing; examples are a tie being worn or not (Fig. 1a), a towel hanging on the wall (Fig. 1b), a missing place setting (Fig. 1c) or the room lights being on or off.

### Sample flicker/movement detection tasks

Participants were asked if a sine wave grating pattern is flickering or steady; or if they are able to tell if a person is waving his hands or keeping them still (e.g., see videos through the link in Supplementary Document).

### Sample direction of motion tasks

Participants were given a scene with objects moving and were asked to report the direction of motion. For example, a soccer ball moving from left to right or right to left; a cursor on the screen moving from left to right, right to left, bottom to top or top to bottom; race cars moving from right to left or left to right, or not moving (e.g., see videos).

### Test administration

Before starting the VR test, participants were given a few practice trials, first using real-world objects (e.g., a detergent bottle, a towel) and then in VR. For real-world practice, they were seated at a table facing a white poster board, similar to the room in the virtual environment, to familiarize themselves with real examples of the virtual scenes they would encounter in the test. First, they were asked to report when the actual room light turns on and off. Then they were presented with a black towel (a real one) and a dark detergent bottle that they had to find on the table, which they could touch with their hands. These three sets of real-world items were shown so that they could understand the context of the study before being introduced to the VR environment. Then, for further practice, the same scenes were presented in the VR headset before starting the test.

At the start of each new activity, participants were shown a demo of each of the *m* alternatives, at the easiest visibility level. After completing the demo, testing began, with participants’ responses recorded. The VR testing order of the 19 items was randomized for each participant. Each item was presented for 3 trials per difficulty level, starting with the easiest level; within each level, alternatives were chosen randomly across trials, with replacement, so the same alternative could be presented more than once.

### Study design

In a prospective design, a heterogeneous group of individuals with ULV between 17 and 91 years of age was recruited for the study. The study was approved by the Johns Hopkins Medicine Institutional Review Board. Informed consent was obtained from all participants before starting the test, and the study procedures conformed to the tenets of the Declaration of Helsinki. A total of 37 individuals participated the study. Recruitment took over 12 months, and testing was conducted in the ultra-low vision lab at the Wilmer Eye Institute (n = 12) and in the participants’ homes (n = 25).

### Data analysis

Responses for each trial were scored as ‘1’ for correct and ‘0’ for incorrect. Scores were then summed across 3 trials so that the highest score for an item would be ‘3’ and the lowest score ‘0’. In other words, raw responses were scored as a success rate.

We were interested in two outcome measures; one, the ‘item measure’ indicating the estimated difficulty of each item,’ and two, the ‘person measure’ indicating the estimated ability of each person. The item measures allow us, for example, to estimate how easy/difficult detecting room light is compared to place settings or direction of motion of cars, along a common scale on which each of these items is located. The difference between person and item measure can be mapped mathematically to the probability the person will respond correctly to the item. This cannot be done by simply comparing accuracy levels using raw scores for each item, because scoring 67% on room light detection in a bright light setting is not the same as scoring 67% on room light detection in a low light setting or detecting a tie with high or low contrast.

Typically, Rasch analysis is used to estimate item and person measures from ordinal response data when differences in performance are due to a latent variable. An example of this is the ULV-VFQ that assesses perceived difficulty in performing activities of daily life using a Likert scale. However, Rasch analysis is technically not applicable when a person’s response is scored “correct” or “incorrect” by someone else such as the test administrator, as in $m$-AFC tasks because chance performance is possible. Rasch analysis applies to rating scale data, but not to our experimental paradigm of $m$-AFC tasks. For these reasons we used the signal detection theory (SDT)-based method described by Bradley and Massof^38^, a latent variable analysis for $m$-AFC tasks, to estimate person and item measures on a relative d prime (${d}^{^{\prime}}$) scale. On this relative ${d}^{^{\prime}}$ scale, the difference between any person measure and item measure represents the estimated ability of that person to perform that $m$-AFC task, measured in relative ${d}^{^{\prime}}$ units.

Unlike in traditional applications of SDT, negative ${d}^{^{\prime}}$ is common (especially for items) on this relative ${d}^{^{\prime}}$ scale because all persons and items are placed on the same scale and not all items are equally difficult. The origin of the relative ${d}^{^{\prime}}$ axis represents chance performance for the average person in the sample. Therefore, many (if not most) items will have a negative ${d}^{^{\prime}}$ because ${d}^{^{\prime}}$> 0 for an item would mean below chance performance for the average person. Negative ${d}^{^{\prime}}$ will also occur for some persons because some persons may have well below average ability. The more traditional use of ${d}^{^{\prime}}$ as a (generally) non-negative measure of person ability is more closely approximated when comparing one person to one item; however, the constraint of placing all persons and items on the same scale will result in some person-item comparisons having negative ${d}^{^{\prime}}$, i.e., a predicted ability less than chance performance. All data were analyzed using the R programs in the paper (https://sourceforge.net/projects/sdt-latent/files/).

## Results

**Figure 2:**
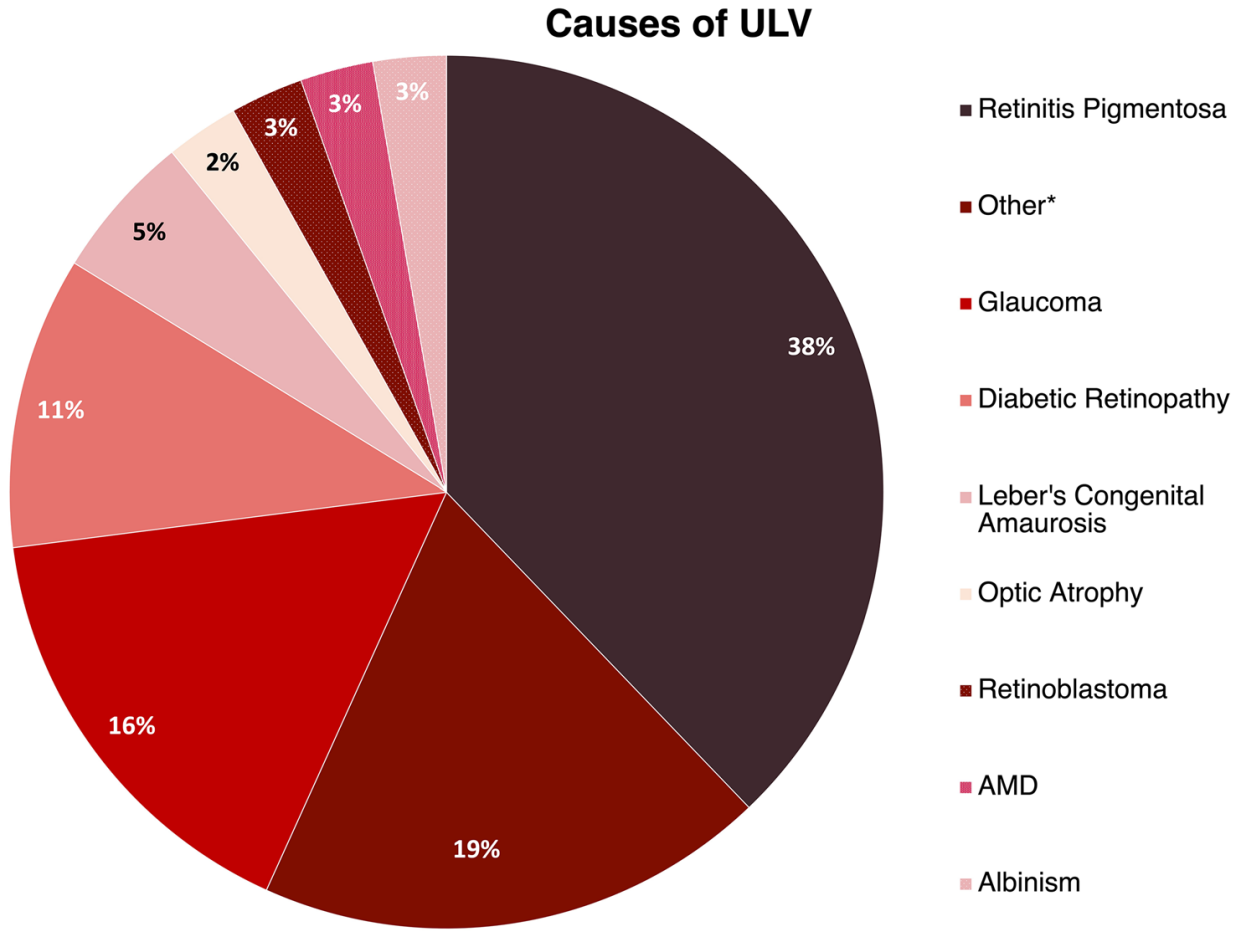
Causes of the ULV among participants in the study. *Other conditions included Achromatopsia, Albinism, Leber’s Congenital Optic Neuropathy, Hydrocephalus, Bardet-Biedl Syndrome CMV Retinitis, Cone-Rod Dystrophy and Microphthalmia.

The distribution of conditions causing ULV are shown in Fig. 2. The conditions causing ULV were heterogeneous, which is representative of the general ULV population. The largest percentage was retinitis pigmentosa (32%). The mean age of the participants was 52.9 years (17 to 91 years) and the mean visual acuity estimated with the Berkeley Rudimentary Vision Test was 3.2 logMAR (2.0 to 3.5 logMAR). Testing took between 1.5 and 2.5 h with an average duration of 2 h. All our participants were able to complete testing in a single session.

### SDT analysis

**Figure 3:**
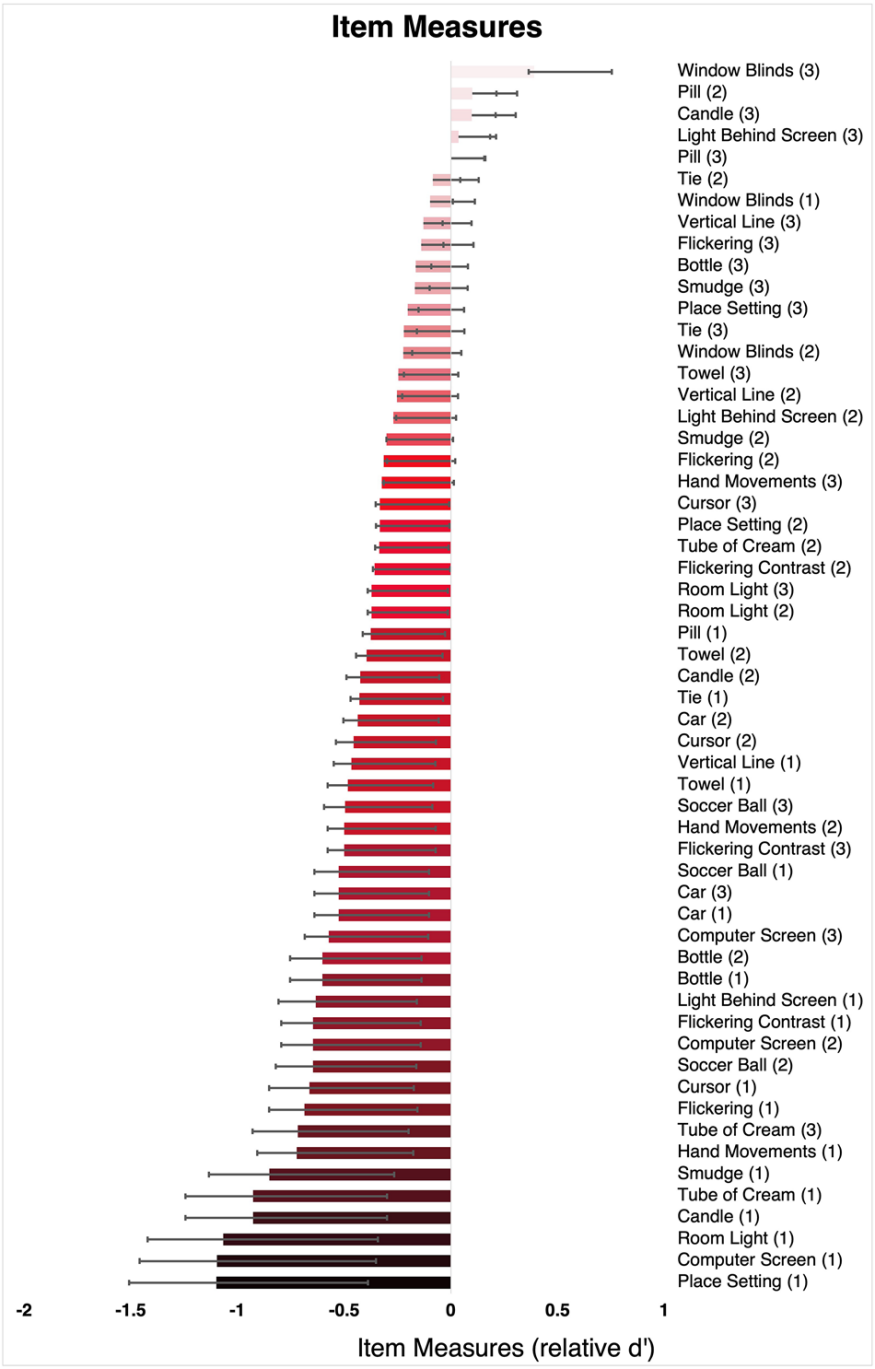
Estimated item measures across different tasks. More positive *d*′ values indicate more difficult items while more negative *d*′ values indicate easier items. Error bars represent the 95% CI of the estimates (see supplement for individual item measures). The number in parenthesis indicates the visibility level: (1) for high, (2) for medium, and (3) for low visibility.

Our first outcome measure was item measure that estimates the item difficulty for all items. Figure 3 shows the distribution of item measures and their 95% CI estimated using SDT for latent variables (see Supplementary Table S2 with individual item measures) ranging from $-$ 1.09 to 0.39 in relative ${d}^{^{\prime}}$ units. There were four items with ${d}^{^{\prime}}>0$, ${d}^{^{\prime}}=0$ indicates chance performance for the average person in the sample and therefore, for these items, performance was below chance level. The easiest items (most negative item measures) included identifying a missing plate under high contrast in a dark room, detecting whether a computer screen was on or off in a dark room and whether bright room lights were on or off. The most difficult items (less negative item measures) included identifying the orientation of white window blinds on a bright background, locating a small grey pill on a black tabletop, and whether a candle was lit in a bright room. As expected, most high contrast items had more negative item measures (i.e., they were easier) while most low contrast items had item measures closer to 0 ${d}^{^{\prime}}$ (i.e., closer to chance performance); medium contrast items were generally in-between. The 95% CI of the estimates, across all items, spanned an interval of 0.58 to 0.74 relative ${d}^{^{\prime}}$ units.

**Figure 4:**
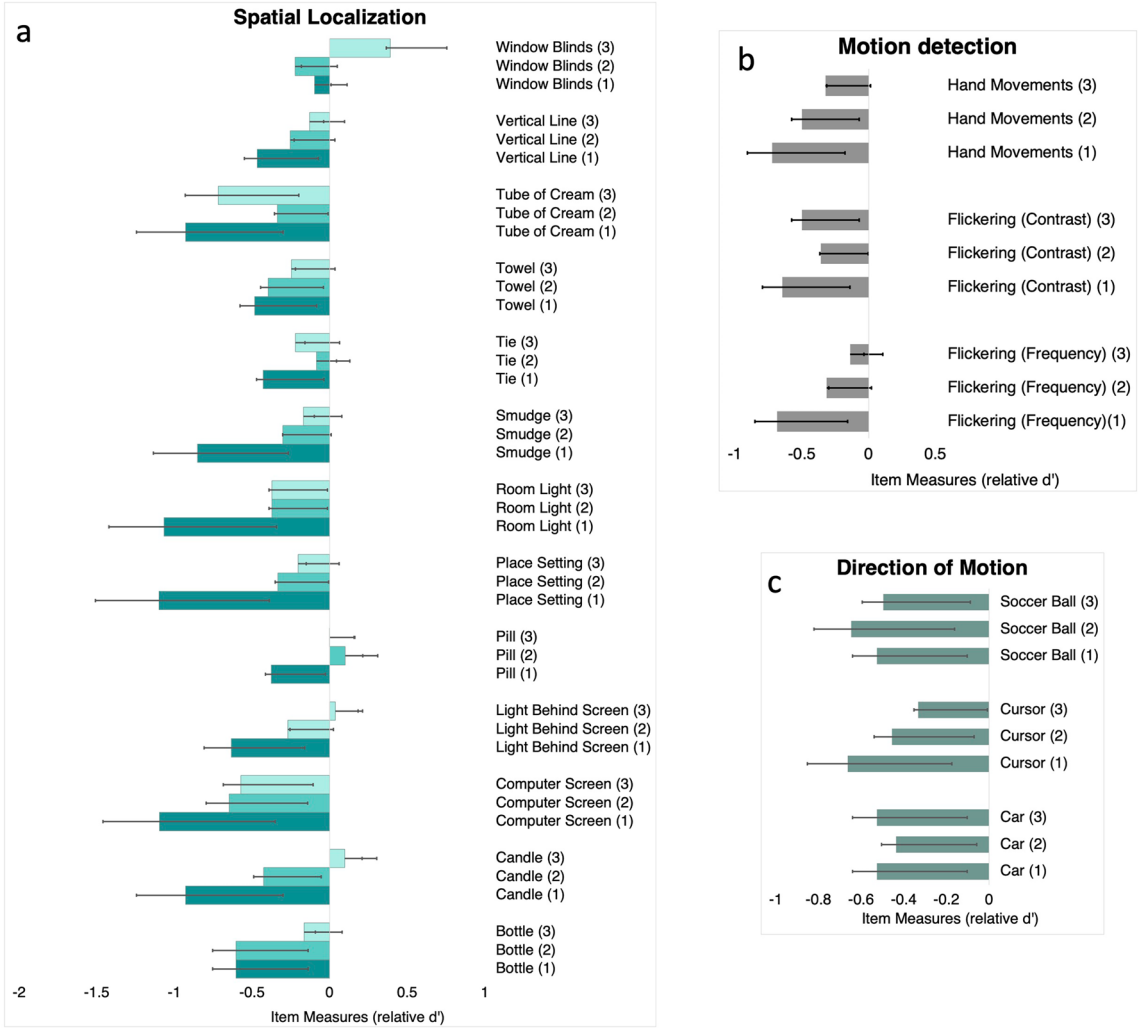
Item measures for spatial localization (**a**), direction of motion (**b**) and motion detection (**c**) tasks. (1) indicates the level with highest visibility, (2) indicates level with medium visibility and (3) indicates the level with least visibility.

As described earlier, our items fell into three categories: spatial localization, motion detection and direction of motion. Figure 4 shows item measure distributions between spatial localization, motion detection and direction of motion. For the spatial localization/detection tasks, item measures became less negative with increasing difficulty with a few exceptions: detecting a tie on a person wearing white shirt, and the Tube of Cream task where the medium levels of contrast were more difficult compared to low levels (Fig. 4a). For the detection of window blinds, medium level was easier compared to high or low contrast levels. Similarly, for motion detection, we found that the item measures shifted to more negative with less difficulty except for the flicker test using contrast variations where medium contrast was more difficult compared to low contrast. For direction of motion tasks this was not true (Fig. 4b,c). Of note, these were items that were closer to chance performance or worse than chance performance in lower contrast levels which could explain these variations.

**Figure 5:**
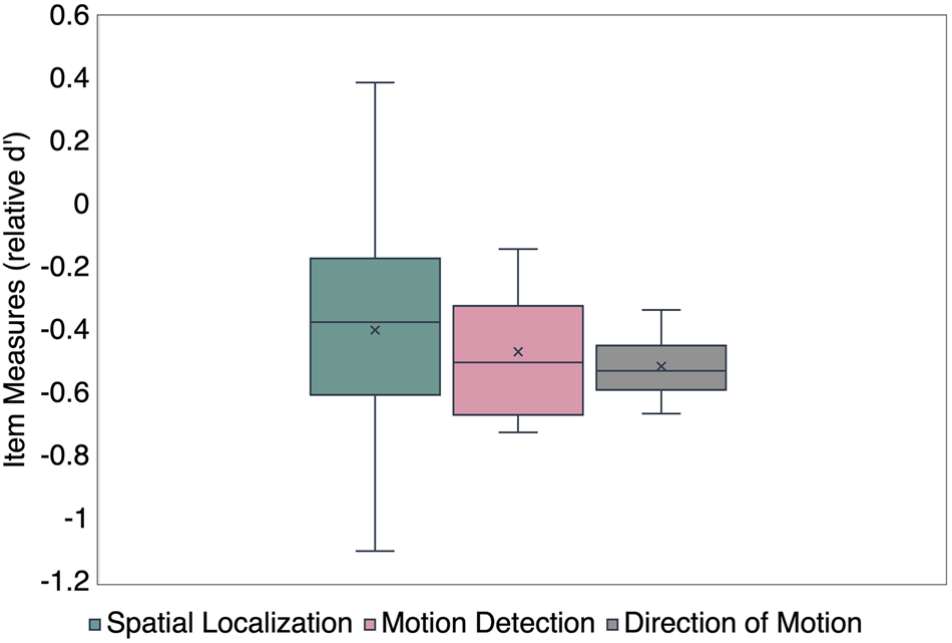
Shows the comparison of item measures across the three categories: spatial localization, motion detection and direction of motion.

A Brown-Forsythe test for equality of means showed that there were no statistically significant differences between the mean item measures in spatial localization/detection ($-$ 0.4 ± 0.35), motion detection ($-$ 0.0.47 ± 0.2) and direction of motion ($-$ 0.5 ± 0.1) tasks (F_(2,54)_ = 0.6; p = 0.54) (Fig. 5).

**Figure 6:**
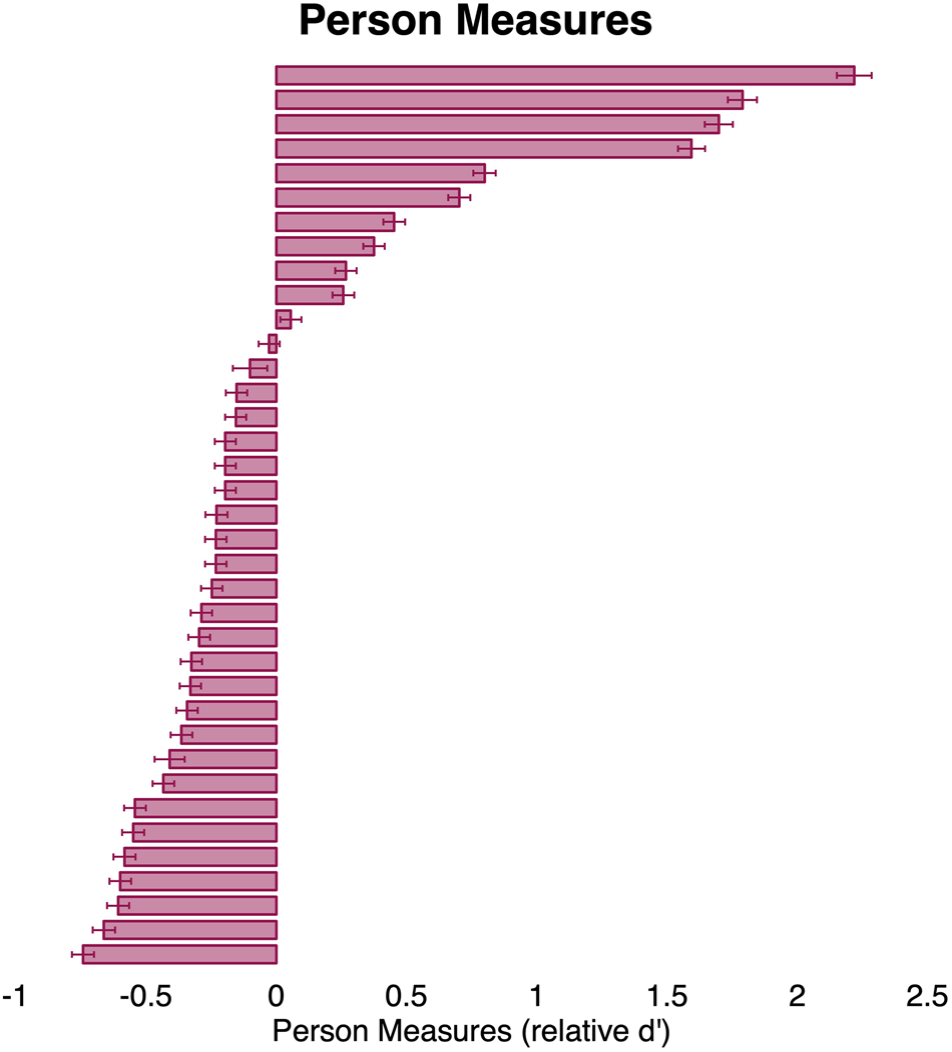
Distribution of person measures across participants. By definition, the sample d’ is zero. Error bars represent the 95% CI of the estimates.

Our second outcome variable was person measure which estimates the ability of individual participants. Figure 6 shows the distribution of person measures among participants with ULV. Person measures ranged between − 0.74 and 2.2 relative ${d}^{^{\prime}}$ (see Supplementary Table S3 for individual person measures). Person measures indicate a person’s ability to perform the tasks and varied widely between participants.

**Figure 7:**
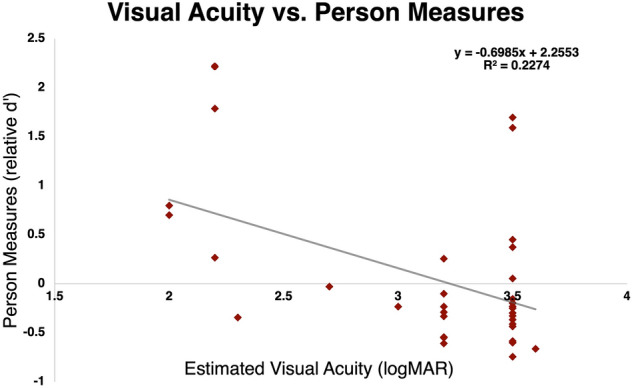
The relationship between visual acuity measured using BRVT and person measures estimated using SDT analysis for individual participants.

Next, we wanted to evaluate how estimated person measure compare to the estimated visual acuity measured using BRVT. Figure 7 shows the relationship between estimated visual acuity measures with the BRVT and person measures. There was a highly statistically significant negative trend towards those with poorer visual acuity having lower person measures (p = 0.002, r^2^ = 0.2, Mean Absolute Error = 0.43), which is expected because a higher person measure and lower logMAR indicate better visual function/ability. The 95% CI for person measures ranged between 0.08 and 0.28 relative *d*′. These could be used to define the minimal clinically important difference (MCID) as a change in person measure (e.g., due to an intervention) outside the 95% CI.

**Figure 8:**
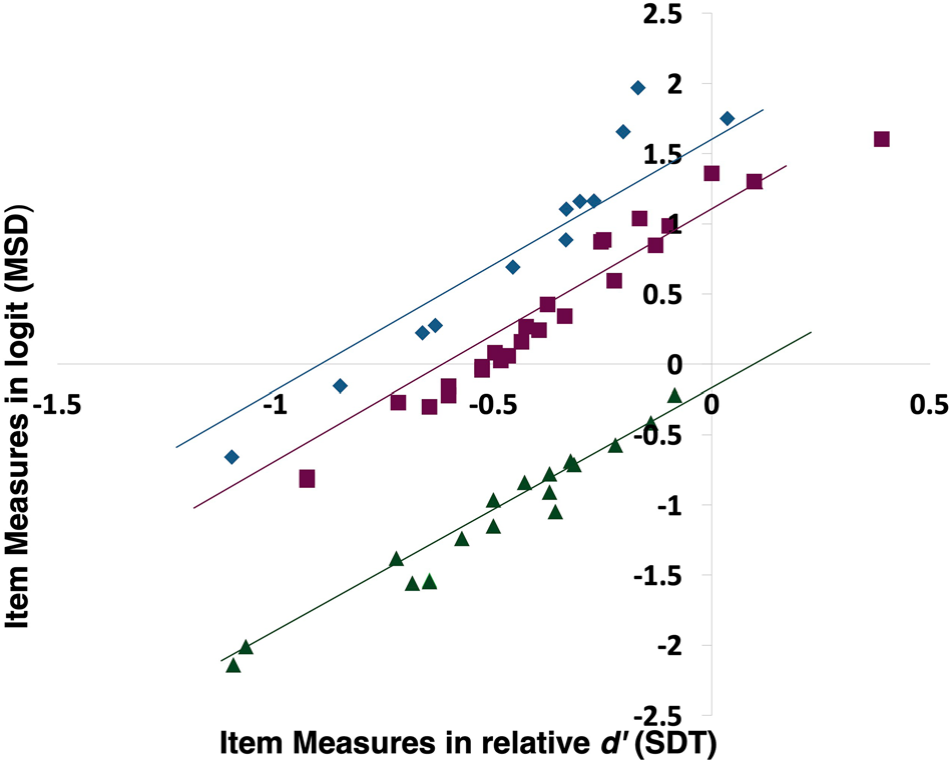
Comparison of item measures estimated using SDT and Rasch analysis. Item measures are stratified according to the number of response alternatives $m$ = 2, 3, and 4 because SDT equates chance performance for all $m$-AFC tasks at ${d}^{^{\prime}}=0$ while Rasch analysis does not. The diamond shape symbols (blue) show $m$-AFC = 4, square shape symbols (pink) show $m$-AFC = 3 and triangle shape symbols (green) show $m$-AFC = 2.

Figure 8 shows the difference between item measures estimated using SDT and Rasch analysis. SDT equates chance performance for all $m$-AFC tasks at ${d}^{^{\prime}}=0$, while Rasch analysis does not, which leads to stratification by the number of response alternatives $m$ when plotting the two sets of item measures against each other. Because chance performance should represent equal difficultly regardless of $m$, we used SDT in this study instead of Rasch analysis. Rasch analysis applies to questionnaires where a person “rates” an item and there is no defined correct or incorrect, while SDT is more appropriate for $m$-AFC tasks.

## Discussion

In this paper, we have shown the development and calibration of a novel visual performance test to assess visual information gathering performance in people with ULV using virtual reality. The number of vision restoration treatments that recruit people with ULV (gene therapy or stem cell therapy) or restore vision to the ULV levels (retinal/cortical prosthesis) is on the rise. However, there is a dearth of standardized tests that can evaluate changes in visual performance following such interventions. Without standard outcome measures, it becomes difficult to compare changes in visual performance among individuals with ULV as well as across different treatment modalities and centers. To address the gap in standardized measures, we developed this set of items based on activities relevant to the ULV population.

In the real world, it is difficult to reproduce the exact conditions for standardized activities between testing rooms. Using a virtual reality platform to present visual information has two important advantages: (1) it gives us the ability to control contrast, luminance, and other test parameters; (2) it provides portability for use in patients’ homes and other non-clinical locations. The latter is particularly important when developing tests for ULV: People with ULV are highly sensitive to changes in visibility and their performance varies significantly based on ambient conditions. Most individuals with ULV also rarely travel independently, which makes it difficult to do extensive functional vision evaluations that may require more than one visit to the clinic. Out of the 37 participants in our study, only 12 were able to travel to our laboratory; all others were tested in their own homes. Another important advantage of using virtual reality is the ability of the examiner to eliminate non-visual (auditory, tactile) cues so that visually guided behaviors can be studied in isolation.

To reiterate, the purpose of creating different visibility levels for each task was to develop a scale for items relating to everyday tasks, ranging from the most difficult to least difficult based on performance by a population with a wide range of ULV. We were able to accomplish this by changing the contrast, luminance, speed, size or frequency across the items and utilize an analysis that still allows us to estimate a level of difficulty for each item that can be compared with all other items, on the same scale. This functional vision assessment is different from a typical psychophysical visual function assessment varying a single physical attribute of the stimulus.

We estimated two outcome measures in this study. The first one, the *item measure,* reflects a scale for item difficulty for the range of items used. Different items can be ordered on this scale to determine the spread of difficulty levels across different items. We have calibrated the items in a representative sample of individuals with ULV, using signal detection theory for latent variable analysis. The 57 activities presented here form an *item bank* from which we can use a subset to estimate person measures in future studies. We found that many item measures, particularly in the middle of the difficulty range, are spaced within 1 SE of each other; even with narrower SEs that will result from future calibration in a larger population of ULV individuals, a certain level of redundancy will remain. Therefore, future studies will be able to use a subset of these items for assessments. It has to be noted that item measures are independent of each other, so removing certain items will not affect the estimates of the measures, although it will increase the SE of the person measure. In situations where precision is not critical, such as routine evaluations in clinical settings^39^, this will reduce the testing time from 1.5–2 h to 20–30 min. The fact that many items have similar item measures means that, during clinical testing, a choice can be made between items of similar difficulty; the choice of items could be changed between sessions or to match the patient’s daily activities, or redundant items could be reserved for training to avoid familiarity in pre-post evaluations.

Furthermore, we showed that the item measures across three difficulty levels showed consistent change with decrease in visibility for variations in contrast, luminance and speed or frequency. People with ULV were more sensitive to changes in contrast and luminance. They also had marginally lower item measures for tasks involving motion detection and direction of motion compared to spatial localization which means that tasks involving motion detection were less difficult compared to those involving spatial localization and detection. Both these findings are important for consideration for vision restoration and rehabilitation where traditionally there has been more focus on spatial localization and detection tasks than on motion detection and direction tasks. The larger effect of contrast and lighting on item measures confirms our previous report that these two visual aspects are critical for the use of remaining sight in people with ULV^35^.

The second outcome, the *person measure,* is an estimate of a person’s visual ability, based on the tasks performed. This is a vital measure that can be used in studies to report changes following various interventions and clinical trials (e.g., gene therapy, stem cell therapy, visual prosthesis, and/or vision rehabilitation programs). Our ULV group was heterogeneous with a wide range of causes and visual abilities that are representative of the ULV population. We found that there was a significant correlation between the estimated visual acuity measurements and *person measures* further confirming the sensitivity of this test in clinical applications to monitor changes in visual ability.

Visual acuity is considered the current gold standard for all vision restoration clinical trials. An improvement of > 2 lines on the ETDRS chart is typically considered the minimum clinically important difference (MCID). However, this may not always reflect improvement in daily living skills^36^. In this study, the relationship between visual acuity and person measures was weak showing that visual acuity is not a good predictor of visual ability as it relates to daily tasks and hence cannot be considered as a proxy for performance measures. We had many participants with the same estimated visual acuity but different person measures. A functional MCID criterion could be defined on the basis of standard errors of the estimated person measures.

The results from this study have many implications beyond clinical testing as virtual reality technology is gaining popularity across different fields of physical medicine and rehabilitation. For example, a recent study reported the use of virtual reality to train orientation and mobility skills in people with severe visual impairment. Participants were given training for street crossing under virtual conditions and were found to have results similar to those who underwent real street training^40^. Virtual reality has also been used in visual rehabilitation of patients with hemianopia who underwent telerehabilitation that showed improved visual performance^41^. VR-based training has also demonstrated benefits in sports medicine, traumatic brain injury, and for eye-hand coordination in trainee surgeons^42^.

One of the potential drawbacks of virtual reality could be cybersickness experienced by users due to conflicting visual and vestibular stimuli. However, no such conflict existed in the immersive environment presented by our stimuli, and none of our subjects reported cybersickness during this study. Another weakness is the inability to compare real-world and virtual world performance on the same tasks by our subjects. However, from other studies that used virtual reality^40,41^ it can be fairly assumed that there is a significant correlation between real-world and virtual world performance. A third possible weakness is that the weight of the VR headset (~ 0.8 kg) could make it hard to conduct prolonged rehabilitation sessions with subjects. This can be resolved by giving them adequate breaks; incidentally, in our experience subjects' visual fatigue, rather than headset weight, was the most common reason for subjects to take breaks. Head mounted devices are constantly evolving and the new headsets are already less bulky; some have an open field of view for mixed reality applications.

Having realized the potential of this assessment for future clinical trials, we are currently developing and validating two additional modules to assess the domains of visual-motor performance and visual wayfinding in people with ULV. Together, these modules will provide insight into the performance of visually guided activities by people with ULV.

## Supplementary Information

Supplementary Information 1.

Supplementary Table S1.

Supplementary Table S2.

Supplementary Table S3.

## Data Availability

All data generated or analyzed during this study are included in this manuscript or as Supplementary Information files.

## References

[1] An analysis for Global Burden of Disease Study (2021). GBD 2019 Blindness and Visual Impairment Collaborators. Trends in prevalence of blindness and distance and near visual impairment over 30 years. Lancet Glob. Health.

[2] Geruschat DR, Bittner AK, Dagnelie G (2012). Orientation and mobility assessment in retinal prosthetic clinical trials. Optom. Vis. Sci..

[3] Sahel JA (2021). Partial recovery of visual function in a blind patient after optogenetic therapy. Nat. Med..

[4] Fernandez E (2021). Visual percepts evoked with an Intracortical 96-channel microelectrode array inserted in human occipital cortex. J. Clin. Investig..

[5] Wood EH (2019). Stem cell therapies, gene-based therapies, optogenetics, and retinal prosthetics: Current State and Implications for the future. Retina.

[6] Ho E, Boffa J, Palanker D (2019). Performance of complex visual tasks using simulated prosthetic vision via augmented-reality glasses. J. Vis..

[7] Stanga PE (2021). Electronic retinal prosthesis for severe loss of vision in geographic atrophy in age-related macular degeneration: First-in-human use. Eur. J. Ophthalmol..

[8] Barnes N (2016). Vision function testing for a suprachoroidal retinal prosthesis: Effects of image filtering. J. Neural Eng..

[9] Karapanos L (2021). Functional vision in the real-world environment with a second-generation (44-Channel) suprachoroidal retinal prosthesis. Transl. Vis. Sci. Technol..

[10] Humayun MS, de Juan Jr E (1998). Artificial vision. Eye.

[11] Humayun, M. S.* et al.* Preliminary 6 month results from the Argus II epiretinal prosthesis feasibility study. In *Annu Int Conf IEEE Eng Med Biol Soc* Vol. 2009, 4566–4568 (2009). 10.1109/IEMBS.2009.5332695 PMC3337704 19963839

[12] Beauchamp MS (2020). Dynamic stimulation of visual cortex produces form vision in sighted and blind humans. Cell.

[13] Oswalt D (2021). Multi-electrode stimulation evokes consistent spatial patterns of phosphenes and improves phosphene mapping in blind subjects. Brain Stimul..

[14] Kuppermann, B., Boyer, D. S., Mills, B., Yang, J. & Klassen, H. J. Safety and activity of a single, intravitreal injection of human retinal progenitor cells (jCell) for treatment of retinitis pigmentosa (RP). In *ARVO2018*, Vol. 59 2987 (Invest Ophthalmol Vis Sci, Honolulu, Hawai, 2018).

[15] Steinkuller PG (2010). Legal vision requirements for drivers in the United States. Virtual Mentor AMA J. Ethics.

[16] Bailey IL, Jackson AJ, Minto H, Greer RB, Chu MA (2012). The Berkeley rudimentary vision test. Optom. Vis. Sci..

[17] Bach M (1996). The Freiburg Visual Acuity test–automatic measurement of visual acuity. Optom. Vis. Sci..

[18] Bach M, Wilke M, Wilhelm B, Zrenner E, Wilke R (2010). Basic quantitative assessment of visual performance in patients with very low vision. Investig. Ophthalmol. Vis. Sci..

[19] Colenbrander A (2005). Visual functions and functional vision. Inter. Congr. Ser..

[20] Colenbrander A (2010). Assessment of functional vision and its rehabilitation. Acta Ophthalmol..

[21] Bennett CR, Bex PJ, Bauer CM, Merabet LB (2019). The assessment of visual function and functional vision. Semin. Pediatr. Neurol..

[22] for Drug HS, for Biologics HS, for Devices HS, Health R (2006). Guidance for industry: patient-reported outcome measures: Use in medical product development to support labeling claims: Draft guidance. Health Qual. Life Outcomes.

[23] Finger RP (2014). Developing the impact of Vision Impairment-Very Low Vision (IVI-VLV) questionnaire as part of the LoVADA protocol. Investig. Ophthalmol. Vis. Sci..

[24] Jeter PE (2017). Development of the ultra-low vision visual functioning questionnaire (ULV-VFQ). Transl. Vis. Sci. Technol..

[25] Rosenman R, Tennekoon V, Hill LG (2011). Measuring bias in self-reported data. Int. J. Behav. Healthc. Res..

[26] Prince SA (2008). A comparison of direct versus self-report measures for assessing physical activity in adults: A systematic review. Int. J. Behav. Nutr. Phys. Act..

[27] Tabrett DR, Latham K (2011). Factors influencing self-reported vision-related activity limitation in the visually impaired. Investig. Ophthalmol. Vis. Sci..

[28] Frank CR, Xiang X, Stagg BC, Ehrlich JR (2019). Longitudinal associations of self-reported vision impairment with symptoms of anxiety and depression among older adults in the United States. JAMA Ophthalmol..

[29] Hróbjartsson A (2013). Observer bias in randomized clinical trials with measurement scale outcomes: A systematic review of trials with both blinded and nonblinded assessors. CMAJ.

[30] Mansfield, J., Ahn, S., Legge, G. & Luebker, A. A new reading-acuity chart for normal and low vision. In *Ophthalmic and Visual Optics/Noninvasive Assessment of the Visual System Technical Digest* Vol. 3, 232–235 (1993).

[31] Legge GE, Ross JA, Luebker A, Lamay JM (1989). Psychophysics of reading. VIII. The Minnesota low-vision reading test. Optom. Vis. Sci..

[32] Tanabe H (2020). Potential roles of MNREAD acuity charts and contrast/glare sensitivity in Ranibizumab treatment of branch retinal vein occlusion. PLoS ONE.

[33] Jonker SMR (2015). Comparison of a trifocal intraocular lens with a +3.0 D bifocal IOL: Results of a prospective randomized clinical trial. J. Cataract Refract. Surg..

[34] Mahmood S (2015). Routine versus as-needed bevacizumab with 12-weekly assessment intervals for neovascular age-related macular degeneration: 92-week results of the GMAN trial. Ophthalmology.

[35] Adeyemo O (2017). Living with ultra-low vision: An inventory of self-reported visually guided activities by individuals with profound visual impairment. Transl. Vis. Sci. Technol..

[36] Ayton L (2020). Harmonization of outcomes and vision endpoints in vision restoration trials: Recommendations from the International HOVER Taskforce. Trans. Vis. Sci. Tech..

[37] Dagnelie G, Geruschat D, Massof RW, Bradley C (2019). Signal Detection Theory (SDT)-based latent variable analysis of ultra-low vision measures with mixed chance levels. Investig. Ophthalmol. Vis. Sci..

[38] Bradley C, Massof RW (2019). Estimating measures of latent variables from m-alternative forced choice responses. PLoS ONE.

[39] Kartha, A., Bradley, C., Sadeghi, R. & Dagnelie, G. Assessing visual potential in ultra-low vision using functional vision tests in virtual reality. In *American Academy of Optometry* (2020).

[40] Bowman EL, Liu L (2017). Individuals with severely impaired vision can learn useful orientation and mobility skills in virtual streets and can use them to improve real street safety. PLoS ONE.

[41] Daibert-Nido M (2021). Case report: Visual rehabilitation in hemianopia patients. Home-based visual rehabilitation in patients with hemianopia consecutive to brain tumor treatment: Feasibility and potential effectiveness. Front. Neurol..

[42] Pieramici DJ, Heimann F, Brassard R, Barteselli G, Ranade S (2020). Virtual reality becomes a reality for ophthalmologic surgical clinical trials. Transl. Vis. Sci. Technol..

