# Supplementary material for: Measuring visual information gathering in individuals with ultra low vision using virtual reality
Source: Sci Rep. 2023 Feb 23;13:3143. doi: 10.1038/s41598-023-30249-z (PMC9950080; doi:10.1038/s41598-023-30249-z)
Supplement: Supplementary file 1 — Supplementary Information 1. [file 41598_2023_30249_MOESM1_ESM.docx]

**Measuring visual information gathering in individuals with ultra-low vision using virtual reality**

Arathy Kartha*^1^; Roksana Sadeghi^2^; Chris Bradley^1^; Chau Tran^3^; Will Gee^3^; Gislin Dagnelie^1^

1. Department of Ophthalmology, Johns Hopkins University School of Medicine, Baltimore, MD
2. Department of Biomedical Engineering, Johns Hopkins University, Baltimore, MD
3. BaltiVirtual Inc., Baltimore, MD


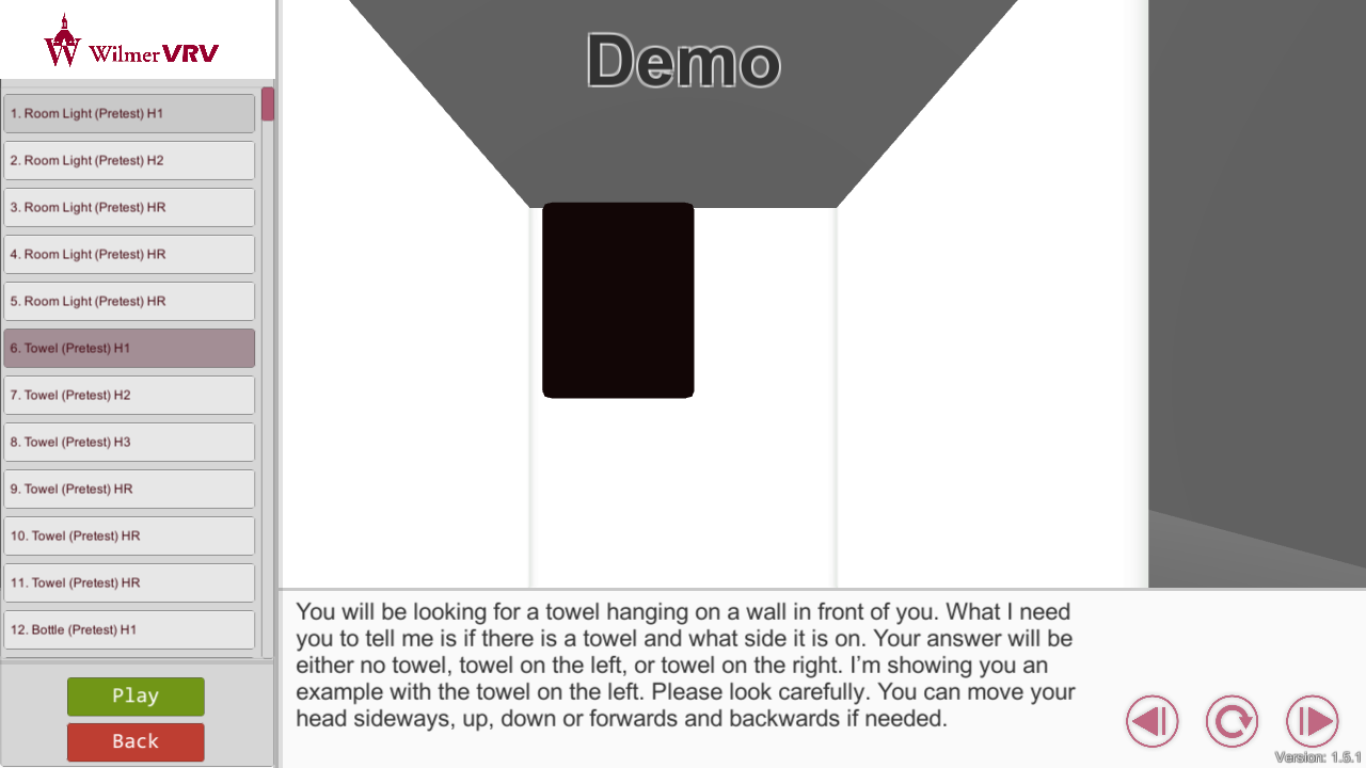


The figure above shows the user interface for the test. On the left of the screen is the playlist that can be customized to the required number of tests. Instructions for each task is given at the bottom of each scene so that they are consistent between participants and testers. On the bottom right, there are three buttons to go forward, backward or replay the items. All figures provided in this document were generated using screenshots of the VR environment (developed by authors CT and WG in Unity) in this study.

The figures below contain description of each task that was part of the test. For all the stationary items, figures are provided for 2, 3 and 4-AFC tasks with the highest visibility level on the left panel followed by medium and low visibility levels. For tasks involving motion, there is a link to the videos provided at the end of this document.

**Room Light ON/OFF**

**2-AFC tasks**

Room showing three different levels of light. Participants were asked to report if the room lights were ON or OFF.

**Tie/No Tie**

Scene showing a man standing wearing tie of different colors. Participants were asked to report if the person was wearing a tie or not.

**Computer Screen ON/OFF**

Scene showing a room with a computer on the table. Participants were asked to report if the computer screen was ON or OFF.

**Locating a Tube of Cream on the Table**

**3-AFC tasks**

**Locating a Detergent Bottle on the Table**

Scene showing a detergent bottle on the table. Participants were asked to report if the bottle was on the left/right/missing.

**Locating a Vertical Line on the Wall**

Scene showing a vertical line on the wall. Participants were asked to report if the line was on the left/right/missing.

Scene showing a tube of cream on the table. Participants were asked to report if the tube was on the left/right/missing.

**Locating a Candle in the Room**

Scene showing a lit candle on the table. Participants were asked to report if the lit candle was on the left/right/missing.

**Locating a Towel on the Wall**

Scene showing a small pill on the table. Participants were asked to report if the pill was on the left/right/missing.

Scene showing a small pill on the table. Participants were asked to report if the pill was on the left/right/missing.

**Locating a Small Pill on the Table**

**Detecting Window Blinds**

Scene showing a window with vertical blinds. Participants were asked to report if the blinds were vertical/horizontal/missing.

**4-AFC tasks**

Scene showing a spot of light in a room. Participants were asked to report if the spot of light was on the upper left/upper right/lower left/lower right.

**Locating a Light Spot**

**Locating a Smudge**

Scene showing a smudge on a mirror on the wall. Participants were asked to report if the smudge was on the upper left/upper right/lower left/lower right.

**Locating a Missing Place**

Scene showing a place setting where one of the plates is missing. Participants were asked to report if the missing plate was at 3 o’clock, 6 o’clock, 9 o’clock or 12 o’clock position.

For tasks involving motion, please see supplementary video files at <https://drive.google.com/drive/folders/1_SCE-P7lEpz3TeAKJYgg3gioSGDVVloH?usp=sharing>
