# Supplementary material for: Measuring visual information gathering in individuals with ultra low vision using virtual reality
Source: Sci Rep. 2023 Feb 23;13:3143. doi: 10.1038/s41598-023-30249-z (PMC9950080; doi:10.1038/s41598-023-30249-z)
Supplement: Supplementary file 2 — Supplementary Table S1. [file 41598_2023_30249_MOESM2_ESM.docx]

**S1. Speed of motion at each difficulty level for the direction of motion tasks**

| **Scene** | **Speed Level** | **Speed (m/s)** |
| --- | --- | --- |
|  | Slow | 0.25 |
| Soccer Ball | Medium | 0.5 |
|  | Fast | 1.0 |
|  | Slow | 2.5 |
| Cursor | Medium | 5 |
|  | Fast | 10 |
| Race Cars | Original race car video that was presented at 1x,2x and 4x | |

Direction of motion tasks involved participants reporting whether an object (soccer ball, race cars) was moving from right to left, left to right or no motion (missing for soccer ball, 3AFC)) and left to right, right to left, top to bottom or bottom to top (cursor, 4AFC).
